# Interaction of rRNA with mRNA and tRNA in Translating Mammalian Ribosome: Functional Implications in Health and Disease

**DOI:** 10.3390/biom8040100

**Published:** 2018-09-26

**Authors:** Amandine Bastide, Alexandre David

**Affiliations:** IGF, CNRS, INSERM, University of Montpellier, F-34094 Montpellier, France; abastide@igf.cnrs.fr

**Keywords:** RNA-RNA interaction, mammalian ribosome, ribosomal RNA (rRNA), transfer RNA (tRNA), messenger RNA (mRNA), RNA modification, hepatitis C virus (HCV), X-linked dyskeratosis congenital

## Abstract

RNA-RNA interaction slowly emerges as a critical component for the smooth functioning of gene expression processes, in particular in translation where the central actor is an RNA powered molecular machine. Overall, ribosome dynamic results from sequential interactions between three main RNA species: ribosomal, transfer and messenger RNA (rRNA, tRNA and mRNA). In recent decades, special attention has been paid to the physical principles governing codon-anticodon pairing, whereas individual RNA positioning mostly relies on ribosomal RNA framework. Here, we provide a brief overview on the actual knowledge of RNA infrastructure throughout the process of translation in mammalian cells: where and how do these physical contacts occur? What are their potential roles and functions? Are they involved in disease development? What will be the main challenges ahead?

## 1. Introduction

Proper folding of RNA through intra- and inter-molecular interactions is essential to the workings of several cellular processes (such as transcription, splicing and translation) that shape gene expression and drive cell fate. This is due to (i) the intimate relationship between RNA structure and function, and (ii) the fact that structured RNAs are central components of essential molecular machines such as the spliceosome, telomerase and ribosome. Base pairing is a major contributor for the stability of both RNA folding and RNA-RNA intermolecular interactions.

**Figure 1:**
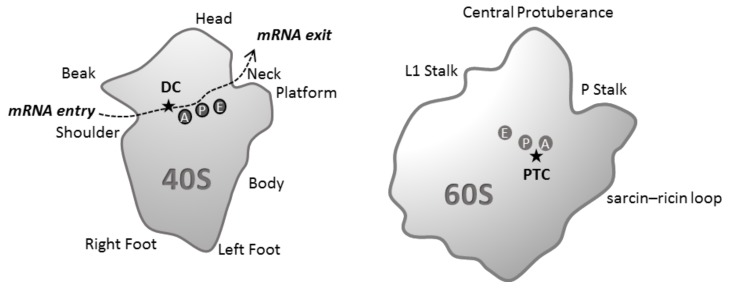
Schematic representation of the structural hallmarks and functional sites of the two mature ribosomal subunits (interface subunit view). The 40S subunit is organized in different structural regions: the body, the head, the shoulder, the beak, and the left and right feet. The 60S subunit structural landmarks are the central protuberance, the L1 and P stalks and the sarcin–ricin loop. Messenger RNA (mRNA) enters the mRNA channel through the mRNA entry site and exits through the mRNA exit site. The mRNA codon is read by its cognate transfer RNA (tRNA) in the decoding center (DC). The peptide bond formation occurs at peptidyl transferase center (PTC). The incoming aminoacyl tRNA enters in the A site, the P site holds the peptidyl tRNA, deacetylated tRNA binds the E site before it dissociates from the ribosome.

RNA-RNA interaction is involved in every step of the ribosome life cycle, from its biogenesis in the nucleus to its cytoplasmic role in protein synthesis. The mature 80S ribosome is composed of two subunits: the small 40S ribosomal subunit contains the 18S ribosomal RNA (rRNA) and 33 ribosomal proteins (RP) and the large 60S ribosomal subunit comprises the 28S, 5.8S and 5S rRNAs and 47 RPs [1]. The 40S subunit is organized in different structural regions: the body (5′ domain of 18S rRNA), the platform (central domain of 18S rRNA), the head (3′ domain of the 18S rRNA), the shoulder, the beak, and the left and right feet (Figure 1).

During the process of translation, messenger RNA (mRNA) enters the ribosome between the head and the shoulder and passes through the mRNA channel. At the ribosome interface the mRNA codon is read by its cognate transfer RNA (tRNA) at the decoding center (DC). The mRNA exits the 40S mRNA channel through the mRNA exit site (between the head and the platform). The 60S subunit displays a crown shape and contains different structural landmarks known as the central protuberance, the two stalks and the sarcin–ricin loop. The peptidyl transferase center (PTC), where peptide bond formation occurs, is located on the interface side of the 60S subunit. The three tRNA binding sites (A, P and E) are located in the cavity between the two ribosomal subunits. The incoming aminoacyl tRNA is recruited in the A site, the binding of the peptidyl tRNA occurs in the P site, and deacetylated tRNA binds in the E site before the release of the discharged tRNA from the ribosome (Figure 1). The whole translation process is cadenced by frequent interactions among mobilized RNA species. Here, we will not cover rRNA interaction with small nucleolar RNA (snoRNA) during ribosome biogenesis (already explained in detail in [2]) nor codon-anticodon base pairing but rather focus on the molecular basis of rRNA interaction with mRNA and tRNA throughout the course of mammalian translation. In addition to summarizing actual knowledge in the field, we seek to discuss the importance of such interactions in fine-tuning translational control of gene expression, both in health and disease.

## 2. Overview of Translation Process

The translation process breaks down into three stages: initiation, elongation and termination. Translation initiation is defined as the process by which the proper start position on the mRNA is identified by the translation machinery and aligned with the anticodon of initiator tRNA. In prokaryotes, the recruitment of the small ribosomal subunit onto the start codon is mediated by base pairing between the mRNA Shine-Dalgarno (SD) sequence and the 3′ end of the 16S rRNA [3]. In contrast, eukaryote translation machinery has considerably evolved, and small ribosomal subunit recruitment does not solely rely on sequence-specific base-pairing interactions between rRNA and targeted mRNA. Instead, the translation initiation process necessitates *cis*-acting elements such as a 5′ cap structure (m^7^GpppN, where N is any nucleotide), a 3′ poly(A) tail and the involvement of at least 11 eukaryotic initiation factors (eIF) [4]. First, the hetero-trimeric eukaryotic initiation factor 4F (eIF4F) complex interacts with the poly(A)-binding protein (PABP) in order to bridge the 5′ and 3′ *cis*-acting elements and circularize the transcript. Second, the 43S initiation complex, which consists of eIF3, eIF1, eIF1A, eIF2-GTP-tRNA_i_^Met^, eIF5 and the 40S ribosomal subunit, is recruited to the m^7^G cap structure of transcripts. The 48S preinitiation complex thus created initiates ribosome scanning toward the start codon. Positioning of the small subunit over the start codon of the mRNA permits the base-pairing interaction between this codon and the anticodon of the initiator tRNA_i_^Met^. This junction is the trigger for a series of events which results in the recruitment of the 60S ribosomal subunit and the assembly of an active 80S with the tRNA_i_^Met^ paired with the start codon in the P site. Next to it, in the A (acceptor) site, the following codon of the open reading frame (ORF) awaits binding of the cognate aminoacyl-tRNA, which will mark the start of elongation [5]. Throughout this stage, amino acid residues that serve as the building blocks of the polypeptidic chain are delivered to ribosomes via tRNA molecules with the assistance of translation elongation factors [6]. Termination occurs when the ribosome encounters a stop codon, which cannot accommodate any tRNA anticodon, hence triggering the translation complex to fall apart. 

RNA-RNA interaction throughout translation process generally refers to codon/anticodon base-pairing, a vital ingredient of the decoding process. Yet, multiple RNA-RNA interactions between ribosomal RNA framework and ribosome RNA substrates, tRNA and mRNA, have been described. Several lines of evidence suggest that these interactions may play a major role in fine-tuning ribosome dynamic and gene expression.

## 3. rRNA-mRNA Base Pairing: Controversy and Exceptions

rRNA-mRNA interaction may principally intervene for recruiting and positioning ribosome during the translation initiation phase. To ascertain mRNA positioning and the fidelity of translation initiation, the start codon is usually adjacent to a characteristic pattern of bases named Kozak sequence ((A/G)CCAUGG) [7] that might serve to slow mRNA scanning and favour start codon recognition. While this pattern makes one think of SD sequence, it does not involve base-pairing with the ribosomal RNA. For this reason, base-pairing between the 18S rRNA remains controversial and, by default, the prerogative of prokaryotic mRNA. Yet, there are always exceptions to every rule: some reports describe cases where base-pairing between eukaryotic mRNA and the 18S rRNA has been proposed as the mechanism for recruiting the ribosome (Table 1). 

The first example of eukaryotic rRNA-mRNA interaction is the ribosomal shunting involving the interaction of the 5′ leader of the mouse Gtx homeodomain mRNA and the 18S rRNA [13]. The authors show that a nine-nucleotides sequence located within the 5′ UTR facilitates translation initiation by base-pairing with complementarity sequences contained within helix 26 (mRNA exit site) of mouse 18S rRNA [8]. Later on, the authors demonstrate that this mRNA-rRNA base-pairing involves the GTX element and drives a SD-like translation initiation process of two cellular mRNAs: *FGF2* and *Gtx* [9]. In 2010, Meng and colleagues reported a SD-like mRNA-rRNA base-pairing interaction between a sequence of the *IGF1R* internal ribosome entry site (IRES) and the helix 23b of the 18S rRNA (within the E-site on the platform of the 40S ribosomal subunit) that may recruit the 40S ribosome and impact IRES translation activity [10]. More recently, the histone H4 (h4) mRNA has been shown to exhibit, shortly after the start codon, a sequence complementary to the 18S rRNA sequence that helps tether the ribosome to the mRNA and favours proper AUG positioning. In addition to 40S interactions with Kozack sequence, base-pairing interactions between a purine-rich sequence in h4 mRNA and a complementary UUUC sequence in the tip of 18S rRNA helix h16 (near the mRNA entry channel) promotes the formation of the 48S initiation complex at the first AUG codon, thus preventing scanning to downstream AUG [11]. All of these studies suggest that rRNA-mRNA interactions could affect translation initiation of a wide number of cellular mRNA. This was further assessed in 2013, when Pánek and colleagues carried out a large scale in silico screening for mRNA-rRNA complementarity across different species and showed that these complementarities only occur between mRNA 5′UTRs and 18S rRNA regions clustered within the 40S ribosomal surface, suggesting that they would be involved in scanning regulation [14]. Finally, one also needs to be cautious that, due to steric constraints, these rRNA-mRNA base-pairings could not be the only regulatory mechanism that contributes to translation regulation: mRNA secondary structures and protein-protein interactions, that induce structural rearrangements, need to be considered in this regulatory process. 

## 4. Does tRNA-rRNA Interaction Set the Pace of Elongation Process?

**Figure 2:**
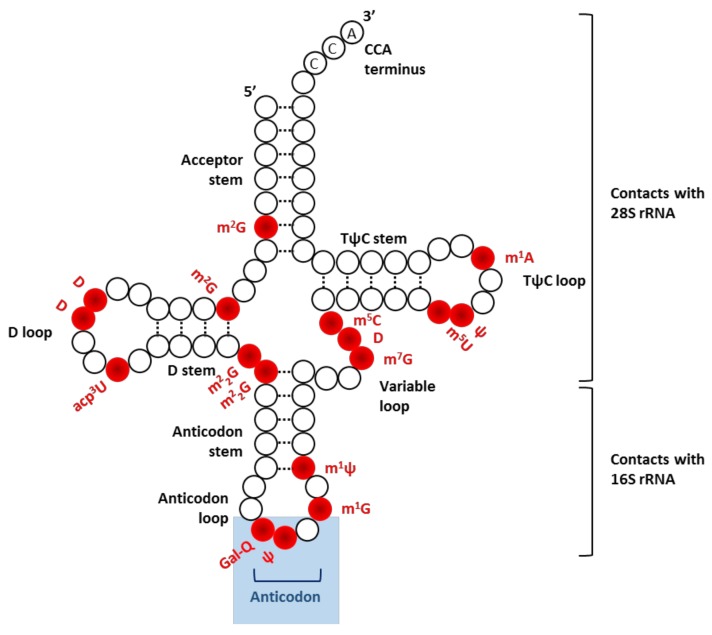
Illustration of secondary structure of tRNA. This cloverleaf shaped structure is due to four base-paired stems, three of them terminating with non-base-paired loops: D loop, anticodon loop, and TψC loop. Nucleotide modifications found in tRNA^Tyr^, one of the most modified tRNA, are annotated in red. Short names of individual modification are given according to the RNA modification databases (e.g., Modomics). Ψ, pseudouridine; D, dihydrouridine; m^1^A, *N*^1^-methyladenosine; m^1^G, *N*^1^-methylguanosine; m^1^ψ, *N*^1^-methylpseudouridine; m^2^G, *N*^2^-methylguanosine, m^2^_2_G, *N*^2^,*N*^2^-dimethylguanosine; m^7^G, *N*^7^-methylguanosine; m^5^C, *N*^5^-methylcytidine; m^5^U, *N*^5^-methyluridine; Gal-Q, galactosyl-queuosine; acp^3^U, 3-(3-amino-3-carboxypropyl)uridine.

Positioning of eukaryotic tRNAs within the translating ribosome has been made possible by technological improvements in cryo-electron microscopy (cryo-EM) [15], X-ray crystallography [16] and site-directed cross-linking [17]. All tRNA molecules share a characteristic secondary structure with three hairpin loops that form the shape of a three-leafed clover (Figure 2). Each tRNA contains an anticodon sequence and serves as an adaptor to connect a cognate mRNA codon with a specific amino acid. Throughout the course of translation, correct functioning of charged tRNAs is achieved via their proper dynamical positioning within the ribosome. This process is mediated by specific interactions not only with mRNA but also with translation factors and ribosomal components [18]. Most particularly, several contacts are established between rRNA and structural elements of tRNAs. In the context of the 48S preinitiation complex, a charged tRNA_i_^Met^ is located in the binding pocket of the 40S subunit where the anticodon stem loop interacts with several helices of the body, the platform and the head of 18S rRNA [18]. Upon start codon recognition and correct codon–anticodon interaction, the acceptor stem of tRNA_i_^Met^ is re-oriented toward the P site in order to promote 60S joining [19] and trigger the elongation phase. During mRNA decoding, the anticodon stem-loop keeps interacting with 18S rRNA while the D and T stem-loop establish contacts with 28S rRNA [11]. It is still unclear whether this multitude of local contacts between tRNA and rRNA may be essential for ensuring the smooth conduct of the translation process. Yet, considering how the functional state of the ribosome dictates tRNA positioning in the A and P sites, their existence is certainly not fortuitous. While charged tRNA share a broadly similar backbone, small details make the difference: each tRNA harbors a unique RNA sequence as well as a distinctive combination of post-transcriptional modifications (see next section). Accordingly, structural data-based studies suggest that tRNA-rRNA interactions within the translating ribosome may vary according to respective features of individual tRNA [15,16,20]. In contrast, a biochemical study performed in prokaryotic 70S ribosome revealed a surprising uniformity of both kinetic and thermodynamic parameters among a set of amino acid-tRNA substrates engaged in the process of first peptide bond formation with initiator tRNA [21]. Yet, it remains to be shown whether the uniform decoding properties may extend to prokaryotic elongating ribosomes, let alone eukaryotic ones.

## 5. The Game Changer: Emerging Role of RNA Modification

Recent re-discovery of post-transcriptional modification of nucleotides has significantly shifted our view of gene expression programs. To date, more than 140 different types of RNA modifications have been reported in all kingdoms of life [22]. They comprise the addition of functional groups (e.g., base or ribose methylation) as well as substitution (e.g., uridine conversion into 4-thiouridine), isomerization (e.g., uridine conversion into pseudouridine, Ψ) and reduction (e.g., conversion of uridine into dihydrouridine). They occur in all types of RNA (tRNA, rRNA, mRNA and ncRNA) and are involved in fine-tuning of RNA biological functions. More specifically, RNA modifications alter/stabilize RNA structure and several of them directly participate in base-pairing [23]. These modifications are often located in strategical positions. For instance, rRNA contain up to 2% RNA modifications which cluster in functional domains of the ribosome: at the decoding center (methylations m^6^_2_A_1781_, m^6^_2_A_1782_ and acetylation ac^4^C_1773_ on helix 45), close to the mRNA channel (ac^4^C_1280_ on helix 34), in the tRNA binding sites (hypermodified pseudouridine m^1^acp^3^Ψ_1191_ and methylation m^7^G_1575_), at the ribosome interface (methylations m^1^A_2142_ and m^5^C_2278_) and the peptidyl transferase center (methylations m^1^A_645_, m^5^C_2870_ and m^3^U_2634_, reviewed in [24]). The remarkable conservation of these modification sites throughout evolution [25] reflects their significance for the smooth execution of mRNA translation [26]. In mRNA, chemical modifications of coding sequence (such as *N*^6^-methyladenosine or 2′-*O*-methylation) may impact the translation process by altering tRNA–mRNA [27] or mRNA–rRNA interactions [28], resulting in altered dynamics of translation elongation. In particular, 2′-*O*-methylation sterically perturbs interactions of universally conserved monitoring bases of the small subunit (G530, A1492 and A1493) with cognate codon–anticodon helices. This results in a higher tRNA rejection during proofreading and ribosome stalling [28]. tRNA is the most heavily modified class among RNA species (Figure 2). Besides the role of these chemical modifications in tRNA architecture and stability, their impact on decoding properties and translation is slowly emerging. In a recent report, authors deleted three of the non-essential tRNA modifiers (trm1, trm10, and abp140) in yeast to study their impact on tRNA usage during translation [29]. They demonstrated that the lack of one of the targeted modifications, m^1^G_9_, altered the tRNA usage in translation while having no impact on tRNA stability. This discovery was made possible by the use of Ribo-tRNA-Seq technology that permits to quantify and identify intra-ribosomal tRNAs and detect some of their modifications. The development of this type of mapping technology will be essential to evaluate whether RNA modifications within individual RNA species—rRNA, tRNA and mRNA—could interplay to fine-tune RNA-RNA interaction and protein expression both in a physiological and pathological context.

## 6. Role of RNA-RNA Interaction in Disease

Little is known about the direct implication of either mRNA-rRNA or tRNA-rRNA interaction in disease onset and progression. Yet, where there is a potential cellular mechanism to divert, there will always be a virus to take advantage of it. Many viruses use IRES mediated cap independent translation to hijack the translational machinery of the host cells for their own translation. In the case of hepatitis C virus (HCV), many studies reported the binding of HCV IRES to ribosomal proteins as well as interaction with the 18S rRNA [12]. This IRES-rRNA interaction involves base-pairing of a CCC triplet in the helix 26 of 18S rRNA with a GGG triplet in the apical loops of the HCV IRES subdomain IIId, essential for the IRES activity. Consequently, targeting this interaction using oligonucleotide masking approaches was proposed as a potential therapy for treating hepatitis C [30]. 

In the same vein, rRNA modification plays a role in selective translation of specific mRNA and/or the use of alternative translation initiation sites. In breast cancer, FBL expression alters rRNA 2′-*O*-methylation (2′-*O*-Me) patterns, triggers changes in translational fidelity and promotes cap independent translation of IRES-containing mRNAs [31]. Strikingly, 2′-*O*-Me plasticity occurs in specific regions involved in intermolecular interactions, such as between tRNA and the A-site [32]. Another abundant rRNA modification consists of isomerisation of uridine into pseudouridine (Ψ) (reviewed in [33]). These modifications participate in rRNA folding and are often located at close proximity to sites involved in ribosome interaction with its ligands, tRNA and mRNA [24]. X-linked dyskeratosis congenital (X-DC) is a rare ribosomopathy characterised by bone-marrow failure disorder and cancer predisposition. This pathology is triggered by mutations of the DKC1 gene which encodes Dyskerin, an evolutionarily conserved enzyme involved in rRNA pseudouridylation. As a result, X-DC impairs both ribosome biogenesis and function [34]. This decrease in rRNA pseudouridylation prevents translation of IRES elements containing mRNAs such as the anti-oncogenes TP53, TP27 and the anti-apoptotic factors BCL-XL and Xiap [35]. N^1^-methyladenosine (m^1^A58) modification of tRNA has also been shown to promote cancer progression through translation reprogramming [36], though the precise underlying mechanism remains unclear. Among other possibilities, it would be interesting to evaluate whether the positively charged m^1^A58 (T loop) may influence large subunit recruitment during the initiation phase and/or tRNA positioning (for instance in respect to 28S rRNA) throughout the elongation phase. Overall, studying RNA-RNA interaction in disease onset and/or progression may be of relevance for understanding their functional role at the molecular level.

## 7. Concluding Remarks

While RNA-RNA interactions are at the heart of ribosome function, several aspects have been largely unexplored, owing primarily to technical and/or model limitations. Yet, acquiring this knowledge is a challenge that must be met in order to comprehend how the ribosome fine-tunes gene expression control. For instance, mRNA-rRNA base pairing could well represent an essential pillar of the “ribosomal filter hypothesis” [37]. This model suggests the existence of ribosome-mediated control of gene expression through specific interactions with a set of mRNAs. The above-mentioned examples—FGF2, GTX and *IGF1R*—are an eloquent illustration of mRNA filtering. While this list is certainly not exhaustive, genetic analysis of mRNA-rRNA base pairing is hampered by the susceptibility of the ribosome to point mutations in 18S rRNA, in particular those that may render the ribosome ineffective. 

On the same trend, tRNA selection inside ribosomes may affect decoding properties of elongating ribosomes, for instance in response to environmental cues [29]. tRNA genes that share the same anticodon, but distinct body sequences are named “isodecoders”. Remarkably, isodecoder genes expression varies widely from one cell type to another [38], which suggests that each cell type may harbor a distinctive expression pattern of tRNA isodecoder transcripts. While this distribution could impact ribosome function in a tissue-specific manner [39], whether isodecoder backbone sequence may regulate tRNA-rRNA interaction and impact the ribosome dynamic remains an open question. 

To further complicate matters, the biological relevance of several mRNA chemical modifications is beginning to take shape: several nucleobase modifications profoundly affect structural principles of RNA base pairing [40] and impact their function. Henceforth, there is a real need to investigate the role of modified bases and their interactions in the context of translation process.

Recent years have been marked by improvement in structural biology technologies (X-ray crystallography, cryo-EM [41] and mass spectrometry of nucleic acid complexes [42]) and the birth of novel sequencing tools (such as Ribo-seq [43], tRNA-seq [44] and nanopore [45]). Their usage will certainly contribute to increase our knowledge on RNA-RNA molecular interaction mechanisms that shape translational control and cell fate in response to central cellular signaling pathways such as extracellular-signal-regulated kinase (ERK) and mammalian target of rapamycin(mTOR).

## Figures and Tables

**Table 1:** Examples of ribosomal-messenger RNA (rRNA-mRNA) base pairing.

Example	Type of Interaction	References
*FGF2* and *Gtx* mRNA	Interaction between nine nucleotides located within the 5′ leader sequence and helix 26 of mouse 18S rRNA.	[8,9]
*IGF1R* mRNA	Near-perfect Watson-Crick base-pairing between Stem2/Loop2 sequence of the IRES and the G961 loop (helix 23b) of the 18S rRNA	[10]
*H4* mRNA	Base-pairing interactions between a purine-rich sequence in *H4* mRNA and a complementary UUUC sequence in the tip of 18S rRNA helix h16 (near the mRNA entry channel) promotes the formation of the 48S initiation complex at the first AUG codon	[11]
HCV IRES	Base-pairing of a CCC triplet in the helix 26 of 18S rRNA with a GGG triplet in the apical loops of the HCV IRES subdomain IIId.	[12]

FGF2, fibroblast growth factor 2; IGF1R, insulin-like growth factor 1 (IGF-1) receptor; HCV, hepatitis C virus; IRES, internal ribosome entry site.

## References

[1] Ben-Shem A., Garreau de Loubresse N., Melnikov S., Jenner L., Yusupova G., Yusupov M. (2011). The structure of the eukaryotic ribosome at 3.0 Å resolution. Science.

[2] Lafontaine D.L. (2015). Noncoding RNAs in eukaryotic ribosome biogenesis and function. Nat. Struct. Mol. Biol..

[3] Yusupova G.Z., Yusupov M.M., Cate J.H., Noller H.F. (2001). The path of messenger RNA through the ribosome. Cell.

[4] Sonenberg N., Hinnebusch A.G. (2009). Regulation of translation initiation in eukaryotes: Mechanisms and biological targets. Cell.

[5] Pestova T.V., Lorsch J.R., Hellen C.U.T., Mathews M.B., Sonenberg N., Hershey J.W.B. (2007). The mechanism of translation initiation in eukaryotes. Translational Control in Biology and Medicine.

[6] Frank J., Gao H., Sengupta J., Gao N., Taylor D.J. (2007). The process of mRNA-tRNA translocation. Proc. Natl. Acad. Sci. USA.

[7] Kozak M. (1999). Initiation of translation in prokaryotes and eukaryotes. Gene.

[8] Chappell S.A., Dresios J., Edelman G.M., Mauro V.P. (2006). Ribosomal shunting mediated by a translational enhancer element that base pairs to 18S rRNA. Proc. Natl. Acad. Sci. USA.

[9] Panopoulos P., Mauro V.P. (2008). Antisense masking reveals contributions of mRNA-rRNA base pairing to translation of *Gtx* and *FGF2* mRNAs. J. Biol. Chem..

[10] Meng Z., Jackson N.L., Shcherbakov O.D., Choi H., Blume S.W. (2010). The human *IGF1R* IRES likely operates through a Shine-Dalgarno-like interaction with the G961 loop (E-site) of the 18S rRNA and is kinetically modulated by a naturally polymorphic polyU loop. J. Cell Biochem..

[11] Martin F., Ménétret J.F., Simonetti A., Myasnikov A.G., Vicens Q., Prongidi-Fix L., Natchiar S.K., Klaholz B.P., Eriani G. (2016). Ribosomal 18S rRNA base pairs with mRNA during eukaryotic translation initiation. Nat. Commun..

[12] Pestova T.V., Shatsky I.N., Fletcher S.P., Jackson R.J., Hellen C.U. (1998). A prokaryotic-like mode of cytoplasmic eukaryotic ribosome binding to the initiation codon during internal translation initiation of hepatitis C and classical swine fever virus RNAs. Genes Dev..

[13] Hu M.C., Tranque P., Edelman G.M., Mauro V.P. (1999). rRNA-complementarity in the 5′ untranslated region of mRNA specifying the Gtx homeodomain protein: Evidence that base-pairing to 18S rRNA affects translational efficiency. Proc. Natl. Acad. Sci. USA.

[14] Pánek J., Kolár M., Vohradský J., Shivaya Valásek L. (2013). An evolutionary conserved pattern of 18S rRNA sequence complementarity to mRNA 5′ UTRs and its implications for eukaryotic gene translation regulation. Nucl. Acids Res..

[15] Valle M., Zavialov A., Li W., Stagg S.M., Sengupta J., Nielsen R.C., Nissen P., Harvey S.C., Ehrenberg M., Frank J. (2003). Incorporation of aminoacyl-tRNA into the ribosome as seen by cryo-electron microscopy. Nat. Struct. Biol..

[16] Ogle J.M., Brodersen D.E., Clemons W.M., Tarry M.J., Carter A.P., Ramakrishnan V. (2001). Recognition of cognate transfer RNA by the 30S ribosomal subunit. Science.

[17] Bulygin K., Malygin A., Hountondji C., Graifer D., Karpova G. (2013). Positioning of CCA-arms of the A- and the P-tRNAs towards the 28S rRNA in the human ribosome. Biochimie.

[18] Lomakin I.B., Steitz T.A. (2013). The initiation of mammalian protein synthesis and mRNA scanning mechanism. Nature.

[19] Yamamoto H., Unbehaun A., Loerke J., Behrmann E., Collier M., Bürger J., Mielke T., Spahn C.M. (2014). Structure of the mammalian 80S initiation complex with initiation factor 5B on HCV-IRES RNA. Nat. Struct. Mol. Biol..

[20] Selmer M., Dunham C.M., Murphy F.V., Weixlbaumer A., Petry S., Kelley A.C., Weir J.R., Ramakrishnan V. (2006). Structure of the 70S ribosome complexed with mRNA and tRNA. Science.

[21] Ledoux S., Uhlenbeck O.C. (2008). Different aa-tRNAs are selected uniformly on the ribosome. Mol. Cell.

[22] Machnicka M.A., Milanowska K., Osman Oglou O., Purta E., Kurkowska M., Olchowik A., Januszewski W., Kalinowski S., Dunin-Horkawicz S., Rother K.M. (2013). MODOMICS: A database of RNA modification pathways—2013 update. Nucl. Acids Res..

[23] Chawla M., Oliva R., Bujnicki J.M., Cavallo L. (2015). An atlas of RNA base pairs involving modified nucleobases with optimal geometries and accurate energies. Nucl. Acids Res..

[24] Sharma S., Lafontaine D.L. (2015). View from a Bridge: A New Perspective on Eukaryotic rRNA Base Modification. Trends Biochem. Sci..

[25] Polikanov Y.S., Melnikov S.V., Söll D., Steitz T.A. (2015). Structural insights into the role of rRNA modifications in protein synthesis and ribosome assembly. Nat. Struct. Mol. Biol..

[26] Decatur W.A., Fournier M.J. (2002). rRNA modifications and ribosome function. Trends Biochem. Sci..

[27] Choi J., Ieong K.W., Demirci H., Chen J., Petrov A., Prabhakar A., O’Leary S.E., Dominissini D., Rechavi G., Soltis S.M. (2016). *N*^6^-methyladenosine in mRNA disrupts tRNA selection and translation-elongation dynamics. Nat. Struct. Mol. Biol..

[28] Choi J., Indrisiunaite G., DeMirci H., Ieong K.W., Wang J., Petrov A., Prabhakar A., Rechavi G., Dominissini D., He C. (2018). 2′-*O*-methylation in mRNA disrupts tRNA decoding during translation elongation. Nat. Struct. Mol. Biol..

[29] Chen C.W., Tanaka M. (2018). Genome-wide Translation Profiling by Ribosome-Bound tRNA Capture. Cell Rep..

[30] Matsuda D., Mauro V.P. (2014). Base pairing between hepatitis C virus RNA and 18S rRNA is required for IRES-dependent translation initiation in vivo. Proc. Natl. Acad. Sci. USA.

[31] Marcel V., Ghayad S.E., Belin S., Therizols G., Morel A.P., Solano-Gonzàlez E., Vendrell J.A., Hacot S., Mertani H.C., Albaret M.A. (2013). p53 acts as a safeguard of translational control by regulating fibrillarin and rRNA methylation in cancer. Cancer Cell.

[32] Erales J., Marchand V., Panthu B., Gillot S., Belin S., Ghayad S.E., Garcia M., Laforêts F., Marcel V., Baudin-Baillieu A. (2017). Evidence for rRNA 2′-*O*-methylation plasticity: Control of intrinsic translational capabilities of human ribosomes. Proc. Natl. Acad. Sci. USA.

[33] Penzo M., Montanaro L. (2018). Turning Uridines around: Role of rRNA Pseudouridylation in Ribosome Biogenesis and Ribosomal Function. Biomolecules.

[34] Heiss N.S., Knight S.W., Vulliamy T.J., Klauck S.M., Wiemann S., Mason P.J., Poustka A., Dokal I. (1998). X-linked dyskeratosis congenita is caused by mutations in a highly conserved gene with putative nucleolar functions. Nat. Genet..

[35] Yoon A., Peng G., Brandenburger Y., Brandenburg Y., Zollo O., Xu W., Rego E., Ruggero D. (2006). Impaired control of IRES-mediated translation in X-linked dyskeratosis congenita. Science.

[36] Macari F., El-Houfi Y., Boldina G., Xu H., Khoury-Hanna S., Ollier J., Yazdani L., Zheng G., Bièche I., Legrand N. (2016). TRM6/61 connects PKCα with translational control through tRNAi^Met^ stabilization: Impact on tumorigenesis. Oncogene.

[37] Kondrashov N., Pusic A., Stumpf C.R., Shimizu K., Hsieh A.C., Ishijima J., Shiroishi T., Barna M. (2011). Ribosome-mediated specificity in Hox mRNA translation and vertebrate tissue patterning. Cell.

[38] Evans M.E., Clark W.C., Zheng G., Pan T. (2017). Determination of tRNA aminoacylation levels by high-throughput sequencing. Nucl. Acids Res..

[39] Ishimura R., Nagy G., Dotu I., Zhou H., Yang X.L., Schimmel P., Senju S., Nishimura Y., Chuang J.H., Ackerman S.L. (2014). RNA function. Ribosome stalling induced by mutation of a CNS-specific tRNA causes neurodegeneration. Science.

[40] Seelam P.P., Sharma P., Mitra A. (2017). Structural landscape of base pairs containing post-transcriptional modifications in RNA. RNA.

[41] Wang H.W., Wang J.W. (2017). How cryo-electron microscopy and X-ray crystallography complement each other. Protein Sci..

[42] D’Atri V., Porrini M., Rosu F., Gabelica V. (2015). Linking molecular models with ion mobility experiments. Illustration with a rigid nucleic acid structure. J. Mass Spectrom..

[43] Ingolia N.T., Ghaemmaghami S., Newman J.R., Weissman J.S. (2009). Genome-wide analysis in vivo of translation with nucleotide resolution using ribosome profiling. Science.

[44] Zheng G., Qin Y., Clark W.C., Dai Q., Yi C., He C., Lambowitz A.M., Pan T. (2015). Efficient and quantitative high-throughput tRNA sequencing. Nat. Methods.

[45] Oikonomopoulos S., Wang Y.C., Djambazian H., Badescu D., Ragoussis J. (2016). Benchmarking of the Oxford Nanopore MinION sequencing for quantitative and qualitative assessment of cDNA populations. Sci. Rep..

